# Mineral Nutritional Yield and Nutrient Density of Locally Adapted Wheat Genotypes under Organic Production

**DOI:** 10.3390/foods5040089

**Published:** 2016-12-20

**Authors:** Sergio Daniel Moreira-Ascarrunz, Hans Larsson, Maria Luisa Prieto-Linde, Eva Johansson

**Affiliations:** Department of Plant Breeding, Swedish University of Agricultural Sciences, SE-230 53 Alnarp, Sweden; sergio.moreira@slu.se (S.D.M.-A.); Hans.Larsson@slu.se (H.L.); Maria.Luisa.Prieto-Linde@slu.se (M.L.P.-L.)

**Keywords:** nutritional yield, minerals, organic agriculture, wheat, stability, adaptability

## Abstract

The aim of the present investigation was to investigate the nutritional yield, nutrient density, stability, and adaptability of organically produced wheat for sustainable and nutritional high value food production. This study evaluated the nutritional yield of four minerals (Fe, Zn, Cu, and Mg) in 19 wheat genotypes, selected as being locally adapted under organic agriculture conditions. The new metric of nutritional yield was calculated for each genotype and they were evaluated for stability using the Additive Main effects and Multiplicative Interaction (AMMI) stability analysis and for genotypic value, stability, and adaptability using the Best Linear Unbiased Prediction (BLUP procedure). The results indicated that there were genotypes suitable for production under organic agriculture conditions with satisfactory yields (>4000 kg·ha^−1^). Furthermore, these genotypes showed high nutritional yield and nutrient density for the four minerals studied. Additionally, since these genotypes were stable and adaptable over three environmentally different years, they were designated “balanced genotypes” for the four minerals and for the aforementioned characteristics. Selection and breeding of such “balanced genotypes” may offer an alternative to producing nutritious food under low-input agriculture conditions. Furthermore, the type of evaluation presented here may also be of interest for implementation in research conducted in developing countries, following the objectives of producing enough nutrients for a growing population.

## 1. Introduction

For the increasing world population, sustainable and adequate food production to meet human needs, while producing food of adequate human nutritional value, is of highest importance [1,2,3,4,5,6,7]. Therefore, a complementary metric of nutritional yield was suggested for agricultural production [2]. Also, the impact of nutrient-dense food on human health has been stressed [8], in line with the need for increased nutrition production per hectare to sustain the world’s population with nutritious food. Nutritional yield and nutrient density in staple crops has been taken into consideration in plant breeding [5,7,9,10,11,12], although the main focus has been higher yield. It is well known that breeding for the traditional metric of yield might imply a reduction in the nutritional value of these staple crops [13,14].

In the present changing environment, with increased fluctuations in weather due to climate change and extreme events such as droughts, floods, hailstorms, and cyclones [15], stability in yield and quality parameters (e.g., nutritional yield) are increasingly important in crops. Therefore, stability analyses of crops will become of higher importance in breeding for future cultivars. Such parameters are especially important in staple crops expected to make up the main part of the daily intake of humans. Locally adapted genotypes are especially important when highly nutritious and organic food is to be produced by cultivation; i.e., local adaptation becomes more important when chemical inputs cannot be used [16,17]. Organic production of cereals constituted 4% of those grown in Sweden in 2009 and the trend has been constantly increasing in recent years [18]. Also, the stability (stable performance of genotypes over production years) of the genotypes as well as adaptability to diverse conditions is of relevance for organic production due to the lack of chemical-based inputs to secure stable yield [16].

High nutrition content in crops adapted to organic agriculture has been identified as one major goal [4,16,19]. Several wheat genotypes have previously been identified as highly nutritious and adapted to the organic production practices prevalent in Sweden [3,20,21]. Nevertheless, the issues of nutritional yield, nutrient density, local adaptation, stability, and adaptability to diverse conditions remain to be evaluated and better understood in organically produced staple crops.

Specifically, two minerals, iron (Fe) and zinc (Zn), have been pointed out as being deficit in the human diet [22]. The World Health Organization has identified these two minerals as the two major minerals contributing to human nutritional deficiency [5]. Over 60% of the world’s population is estimated as Fe-deficient and over 30% as Zn-deficient [11,12,23]. Furthermore, these deficiencies are not limited to the developing world but are also present in the developed world [24]. As summarized from literature in the Nordic Nutrition Recommendations 2012, Fe deficiency was found in 18%–26% of Swedish females between the ages of 15 and 21. In general, 10%–22% of Nordic women of childbearing age are Fe-deficient [25]. Therefore, the target to increase the content of the two micronutrients has been prioritized in wheat breeding [5,7,9,26,27,28].

Two other minerals, copper (Cu) and magnesium (Mg), are considered as essential trace elements for human health and lower intake than recommended has been reported in populations both from developing and developed countries [7,9,11,12,29,30]. Mg is one predominant mineral of cereal grains [30], and in fact whole grain cereals are considered one of the main sources of dietary Mg [25]. The intake of Cu and Mg is above the recommended levels in Nordic countries [25]. However, decreasing content of both minerals has been reported in higher-yielding modern wheat cultivars [3,12,13,14].

The present study aimed to increase the understanding of how high nutritional yield and nutrient density can be obtained in organically grown wheat. Therefore, yield and concentration of the four mentioned minerals (Fe, Zn, Cu, and Mg) were evaluated in organic wheat grown over three years in Sweden. The wheat was evaluated for nutritional yield and nutrient density of these four minerals. Another aim was to better understand the presence of local adaptation among wheat genotypes. Thus, the stability and adaptability of yield and nutritional yields, as well as the nutritional value of the genotypes, were evaluated and genotypes were ranked according to their performance in terms of nutritional yield.

## 2. Materials and Methods

### 2.1. Site Characteristics, Field Layout, and Plant Material

A total of 19 winter wheat genotypes were selected to be grown during three years, under organic conditions, in the locality of Ekhaga, Sweden (59°49.9′ N, 17°48.4′ E). The soil characteristics of this locality have been described as: soil pH (measured in water) 6.0–6.2 (from multiple soil samples), organic matter concentration 5%, and clay concentration 50% [3]. Farmyard manure was applied to the soil following application procedures of the whole Ekhaga farm, which has been under organic agriculture conditions since 1990. Since 2004, no manure at all has been applied to the fields used in the present investigation. The 19 genotypes were grown in a completely randomized design with two replications during the three winter production seasons between 2011 and 2014. Plot size of 24 m^2^ with a harvest size of 23 m^2^, planting density of 200 kg·ha^−1^ during the first year with an adjustment to 400 kernels/m^2^ for year two and three, and sowing dates of 17 September 2011, 25 September 2012, and 16 September 2013, were applied. No weed control was used as competition with weeds was another aim (although not of the present manuscript) to evaluate in this wheat material. Each genotype belonged to one of the following six genotype groups: Selections, Old cultivars, Primitive wheat, Spelt, Landraces, and Cultivars. These genotype groups have been described previously [31]. Briefly, Selections consist of genotypes selected from old varieties for organic production, Old cultivars encompass varieties evolved between 1900 and 1960, Primitive wheat includes Einkorn (*Triticum monococcum*) and Emmer (*T. dicoccum*) wheat, Spelt comprise spelt varieties and spelt selections, Landraces consist of wheat landraces traditionally used in organic farming, and Cultivars include varieties evolved after 1970.

### 2.2. Weather Data

Climate conditions recorded during the trials are shown in Table 1. The weather data for autumn and winter describe the conditions from the sowing period of the winter wheat in the autumn of 2011, 2012, and 2013 until the overwintering period that these seedlings underwent (Table 1). The data for spring and summer illustrate the conditions from the beginning of the vegetative growth period until the harvest in the summer of 2012, 2013, and 2014, when the winter wheats were harvested (Table 1). The data for the months of May, June, and July of each year are shown in order to emphasize the conditions during the main growth period for the studied wheat genotypes (Table 1).

### 2.3. Preparation of Samples

After harvest, grain samples were air dried in order to measure the yield at 13% moisture concentration. Subsequently, samples were milled to wholemeal flour using a laboratory mill (Yellow line, A10, IKA-Werke, Staufen, Germany). The flour samples were dried overnight at 40 °C before weighing. To approx. 500 mg of each dried sample, 10 mL of concentrated nitric acid (HNO_3_) were added prior to digestion. The digestion was performed in a microwave digestion system (Microwave Labstation Mars 5, CEM Corporation, Mathews, NC, USA). After digestion, sample volumes were adjusted to 100 mL with MilliQ water [3].

### 2.4. Determination of Mineral Concentration and Nutritional Yield

Concentrations of Fe, Zn, Cu, and Mg in the samples were determined in duplicate by inductively coupled plasma atomic emission spectrometry (ICPAES; OPTIMA 3000 DV, PerkinElmer, Upplands Väsby, Sweden) [3]. The analyses were conducted at the ICP laboratory of the Department of Ecology, Lund University following standard procedures for calibration and blanks.

Nutritional yield, expressed as the number of adults that can fulfill 100% of their daily recommended intake needs with one hectare of wheat per year (adults ha^−1^·year^−1^), was calculated based on the recorded yield and the measured mineral concentration, as detailed elsewhere [2]. For this, data on yields at 13% moisture concentration were employed. Yield after dehulling is normally corrected for hulled genotypes [32]. Here, yields were estimated as 75% of hulled grain for spelt and primitive genotypes. The daily recommended intake (level of intake sufficient for the needs of 97% of individuals in an age- and sex-specific population group) values applied were 12 mg·day^−1^ for Fe, 8 mg·day^−1^ for Zn, 0.9 mg·day^−1^ for Cu, and 315 mg·day^−1^ for Mg [25]. Daily recommended intake values used here for Fe, Zn, and Mg were averages of values recommended for adult men and women.

### 2.5. Statistical Analysis

In order to explain the proportion of the contribution of variation by the used environments and genotypes on the evaluated parameters, yield, and nutritional yield of Fe, Zn, Cu, and Mg, regression analyses was carried out [33]. To evaluate the influence of genotype and environment, the data were analyzed by ANOVA using the R language [34]. Clearly significant (*p* < 0.05) genotype by environment interactions were detected by the ANOVA, though the environmental variation was the result of different cultivation years. However, it is well known from a range of studies including both different plant material and different compounds of analyzes that cultivation year is a major contributor to environmental impact, often comparable in magnitude to other types of environmental impacts e.g., site variation, but also to genotypic variation [35,36,37,38,39]. When genotype by environment interactions are present, the Additive Main effects and Multiplicative Interaction (AMMI) model is suggested as an efficient statistical method to analyze crop yield and is therefore one of the most widely used methods [40,41,42,43]. The AMMI model incorporates an additive portion separated from interaction by ANOVA and a multiplicative part provided by the principal component analysis (PCA). Thus AMMI starts with an ANOVA analysis to compute the genotype and environmental additive effects. Thereafter, PCA is calculated to analyze non-additive interaction effects. The predictive accuracy of AMMI using two replicates has been shown to be similar to mean value comparisons using five replicates [43]. The AMMI analyses have been found applicable to all crops and in all environments [44], where an environment is considered as a particular site-year combination. In the present investigation, three environments have been used. From the AMMI analysis biplots can be built, showing the principal additive effects of the genotype and environment, e.g., the yield, on the horizontal axis and the multiplicative effects of the genotype by environment interactions, i.e., PC1, on the vertical axis. PC1 values indicate stability of the genotype, a higher stability the closer to 0 the PC1 value of the genotype is. Here AMMI analysis was carried out for each of the parameters (yield and nutritional yield) of Fe, Zn, Cu, and Mg, respectively, using the R package “Agricolae” [45].

In order to determine the genotypic value and its stability and adaptability, the BLUP (Best Linear Unbiased Prediction) procedure was employed [46,47] using the R package “lme4” [48]. Genotypes were ranked according to the values of the harmonic mean of relative performance of genotypic values (HMRPGV), which implies a simultaneous ranking by yield, stability, and adaptability to adverse conditions within a specific environment. The values of the harmonic mean of genotypic values (HMGV) illustrate yield genotypic value and stability; the relative performance of genotypic values (RPGV) represents the adaptability of yield to unfavorable conditions [47].

In order to optimize the selection of the genotypes with the highest Fe, Zn, Cu, and Mg nutritional yield simultaneously, two different selection indexes were calculated: Elston’s multiplicative index (EMI) [46,49] and Baker’s desired gains index (BDGI) [46,50]. EMI was calculated using the lowest average values among the 19 genotypes across the three years as the lowest acceptable limit (LAL) values for each nutritional yield, as described previously [39]. Thus, the LAL values were 28 adults ha^−1^·year^−1^ for Fe nutritional yield, 40 adults ha^−1^·year^−1^ for Zn nutritional yield, 50 adults ha^−1^·year^−1^ for Cu nutritional yield, and 25 adults ha^−1^·year^−1^ for Mg nutritional yield. BDGI was calculated assuming a desired nutritional yield of 50 adults ha^−1^·year^−1^ for every mineral studied. This value was selected under the following considerations: The overall nutritional yields observed in the 19 genotypes studied were 39 adults ha^−1^·year^−1^ for Fe, 46 adults ha^−1^·year^−1^ for Zn, 63 adults ha^−1^·year^−1^ for Cu, and 44 adults ha^−1^·year^−1^ for Mg. The overall mean concentration of Fe and Zn in the population of 19 genotypes studied was 43 mg·kg^−1^ and 36 mg·kg^−1^, respectively. The HarvestPlus Challenge Program (www.harvestplus.org) has shown that an increase of 25 mg·kg^−1^ for Fe concentration, and 10 mg·kg^−1^ for Zn concentration in grains, are to be expected in order to have an impact of breeding and selection on human health [51,52]. It has even been suggested that a doubling of Fe and Zn concentrations would be appropriate and achievable for cereal grains [12]. For the overall mean yield of the 19 genotypes evaluated (3959 kg·ha^−1^) to remain unchanged, such increases would equate to nutritional yields of 62 adults ha^−1^·year^−1^ for Fe and 63 adults ha^−1^·year^−1^ for Zn in the first case, and of 79 adults ha^−1^·year^−1^ for Fe and 98 adults ha^−1^·year^−1^ for Zn in the latter case. The value of 50 adults ha^−1^·year^−1^ for both nutritional yields seems a conservative increase goal for these two minerals and the same value was used for Cu and Mg so that no unintentional decrease would be caused by selection for Fe and Zn. Thus, in order to get nutritional yields of 50 adults ha^−1^·year^−1^ for the four minerals through selection, the desired gains used for the calculations were 11 adults ha^−1^·year^−1^ for Fe nutritional yield (increase from 39 to 50 adults ha^−1^·year^−1^), four adults ha^−1^·year^−1^ for Zn nutritional yield (increase from 46 to 50 adults ha^−1^·year^−1^), zero adults ha^−1^·year^−1^ for Cu nutritional yield (neither increase no increase from 63 adults ha^−1^·year^−1^), and six adults ha^−1^·year^−1^ for Mg nutritional yield (increase from 44 to 50 adults ha^−1^·year^−1^).

## 3. Results

### 3.1. Precipitation and Temperature during the Trials

A difference of 0.6 °C was observed between the average autumn–winter mean temperatures during the first year (2.3 °C) and the third year (2.9 °C). However, during the second year, a markedly lower average mean temperature (−0.3 °C) was observed. For the spring–summer period, differences of 1 °C or less were observed among the three years. The highest average spring–summer mean temperature corresponded to the third year (13 °C), and the lowest corresponded to the first year (12 °C; Table 1).

Regarding the degree days with base 10 °C, the year with the highest number of degree days was the second year (883 days in total, 554 days between May and July), followed by the third year (869 days in total, 519 days between May and July). The first year had the lowest number of degree days (727 days in total, 412 days between May and July; Table 1).

In relation to precipitation, the first year accumulated a total of 635.5 mm between autumn and summer, while the second year accumulated 422.5 mm, and the third year accumulated 572.0 mm. The same pattern was observed in the accumulated precipitation during the main growth period between May and July. During the first year, the accumulated precipitation during this period was 208.0 mm, and during the second and third years it was 74.5 mm and 140.0 mm, respectively (Table 1).

### 3.2. Average Yield and Nutritional Yield across Years

The genotypes in the present study showed large variation in average yield and nutritional yield (Table 2). The genotype with the highest average yield across the three years was the old cultivar Starke (5307 kg·ha^−1^), followed by the old cultivar Svale (4912 kg·ha^−1^), the landrace Jacoby (4595 kg·ha^−1^), and the old cultivar Odin (4572 kg·ha^−1^). The lowest average yields corresponded to the spelt genotypes Oberkulmer (3543 kg·ha^−1^) and Speltvete Gotland (2777 kg·ha^−1^), and the primitive genotypes Svart emmer (2525 kg·ha^−1^) and *T. monococcum* (2027 kg·ha^−1^; Table 2). No significant differences in yield or nutritional yield were seen in the present investigation, most likely due to the limited number of genotypes from the various genotype groups.

The genotype with the highest average Fe nutritional yield was the old cultivar Svale (54 adults ha^−1^·year^−1^), followed by the old cultivar Hansa brun (46 adults ha^−1^·year^−1^), the old cultivar Walde (44 adults ha^−1^·year^−1^), and the spelt Oberkulmer (44 adults ha^−1^·year^−1^; Table 2). The old cultivars Walde (52 adults ha^−1^·year^−1^) and Starke (52 adults ha^−1^·year^−1^), and the landrace Jacoby (51 adults ha^−1^·year^−1^) had the highest average Zn nutritional yield across the three years. These were followed by the spelt Oberkulmer (49 adults ha^−1^·year^−1^; Table 1). The highest Cu nutritional yield was found for the old cultivar Odin (79 adults ha^−1^·year^−1^), followed by the old cultivars Svale (75 adults ha^−1^·year^−1^) and Starke (74 adults ha^−1^·year^−1^), and the landraces Borstvete Gotland (71 adults ha^−1^·year^−1^) and Jacoby (70 adults ha^−1^·year^−1^; Table 2). The genotype with the highest average Mg nutritional yield across the three years was the old cultivar Starke (56 adults ha^−1^·year^−1^), followed by the old cultivars Svale (53 adults ha^−1^·year^−1^) and Aura (50 adults ha^−1^·year^−1^), the landrace Jacoby (50 adults ha^−1^·year^−1^), and the cultivar Ure (48 adults ha^−1^·year^−1^; Table 2).

An additional correlation analysis (Table 3) resulted in highly significant (*p* < 0.01) correlation coefficients that ranged between 0.71 (between Fe nutritional yield and Cu nutritional yield) and 0.95 (between yield and Mg nutritional yield) for the five variables studied (yield; and Fe, Zn, Cu, and Mg nutritional yield).

### 3.3. Stability Analysis of Yield

**Figure 1:**
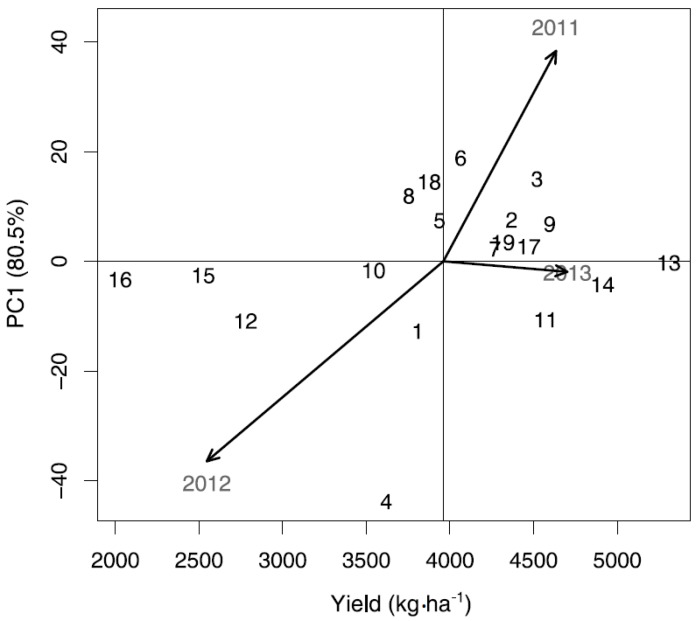
Additive Main effects and Multiplicative Interaction (AMMI) biplot for yield data showing yield (kg·ha^−1^) versus the first principal component (PC1) score of the 19 winter wheat genotypes and the three years of production in the locality of Ekhaga. PC1 explains 80.5% of the genotype by year interaction variance. Genotypes are represented by the ID numbers from Table 1. Years of production are designated by the year of harvest. The vertical axis indicates the overall average yield (kg·ha^−1^) across the three years of production. Genotypes closer to the horizontal axis (score 0 on PC1) had a relatively more stable yield across the three years.

Regression analyses showed that variation in yield, Cu nutritional yield, and Mg nutritional yield was determined to a higher degree by the genotype than by the environments used in the present study. However, variation in Fe and Zn nutritional yield was explained more by the environment than by the genotypes used, thereby indicating the importance of various years for these parameters. Genotype × environmental variation was found for all parameters investigated, allowing us to use the AMMI analyses to evaluate genotype stability for environmental changes in a specific locality. The first principal component, based on genotype by year interactions, of the AMMI stability analysis of yield explained 76.3% of the variance. For genotypes with above-average yield (>4152 kg·ha^−1^), AMMI stability analysis of yield indicated the old cultivars Starke, Hansa brun, and the cultivar Ure to be more stable (PC1 close to zero; −0.30, 2.26, and 2.69, respectively) than the other above-average genotypes studied (Figure 1). The spelt Oberkulmer and the primitive genotype Svart emmer were more stable than the other below-average yielding genotypes, with PC1 of −1.70 and −2.59, respectively (Figure 1). Figure 1 also suggests that yield stability was more prevalent in above-average yielding genotypes than in below-average yielding genotypes. The second year of production (2013) showed a below-average and unstable yield (score of −40.48), while the first and third years resulted in above-average yields. The first year was the most unstable of these (score of 42.60) and the third year was the most stable (score of −2.12) regarding yield.

### 3.4. AMMI Stability Analysis of Fe, Zn, Cu, and Mg Nutritional Yield

**Figure 2:**
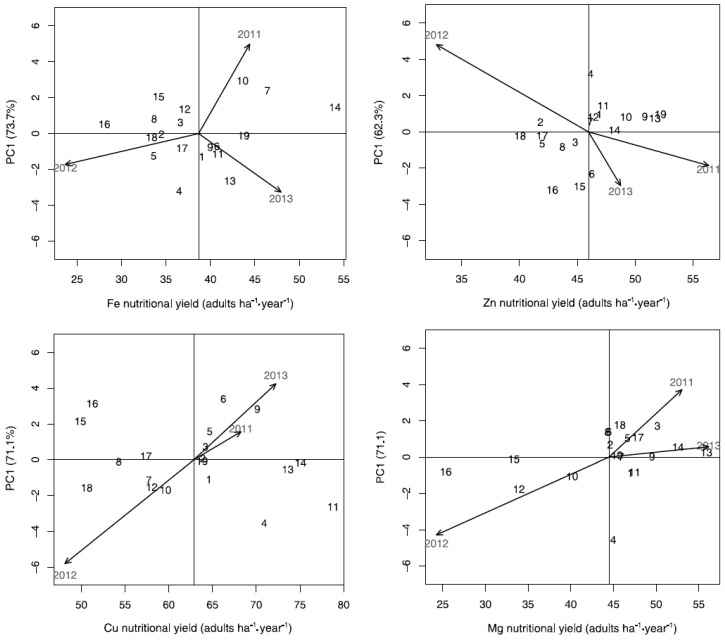
AMMI biplots of nutritional yield showing nutritional yield scores (adults ha^−1^·year^−1^) for (**a**) Fe, (**b**) Zn, (**c**) Cu, and (**d**) Mg versus the first principal component (PC1) score of the 19 winter wheat genotypes and the three years of production in the locality of Ekhaga. The first principal components (PC1) explain 73.7%, 62.3%, 71.1%, and 71.1% of the genotype by year interaction variance of Fe, Zn, Cu, and Mg nutritional yield, respectively. Genotypes are represented by the ID numbers from Table 1. Years of production are designated by the year of harvest. The vertical axis indicates the overall average nutritional yield (adults·ha^−1^·year^−1^) across the three years of production. Genotypes closer to the horizontal axis (score 0 on PC1) had a relatively more stable nutritional yield across the three years.

For genotypes with higher than average Fe nutritional yield, the AMMI stability analysis showed that the old cultivar Walde was the most stable (score of −0.12 on the first principal component) in relation to other genotypes, followed by the old cultivar Ertus (score of −0.71) and the landrace Jacoby (score of −0.76). Among the genotypes with lower than average Fe nutritional yield, the old cultivar Aros was the most stable (score of −0.04), followed by the old cultivar Vakka (score of −0.21), the primitive *T. monococcum* (score of 0.51), and the old cultivar Aura (score of 0.62; Figure 2a). Higher stability scores were associated with genotypes around and below the overall Fe nutritional yield mean, and less so with genotypes with the highest Fe nutritional yields. The second year of the study was shown as the one with the lowest but most stable Fe nutritional yield (score of −1.91). The first and third years of study had above-average Fe nutritional yields, with the third year being the most stable (score of −3.62; Figure 2a).

Regarding the genotypes with higher than average Zn nutritional yield, the old cultivar Svale appeared as more stable (score of 0.10) than the other genotypes, followed by the old cultivars Hansa brun (score of 0.56) and Starke (score of 0.74). The most stable among the lower than average genotypes were the cultivar Ure (score of −0.20) and the old cultivar Vakka (score of −0.22), followed by the old cultivars Aros (score of 0.54), Aura (score of −0.57), and Erbe (score of −0.65; Figure 2b). The second year of the study had lower than average and the least stable Zn nutritional yield (score of 5.35). Conversely, the first and third year had above-average Zn nutritional yields, and the year with the most stable Zn nutritional yield was the first year (score of −2.06; Figure 2b).

The genotypes with most stable higher than average Cu nutritional yield were the old cultivars Aros (score of 0.07), Walde (score of −0.08), Svale (score of −0.17), and Starke (score of −0.54). Among the genotypes with lower than average Cu nutritional yield, the most stable ones were the old cultivar Holger (score of −0.11) and the cultivar Ure (score of 0.20; Figure 2c). Stability was most associated with genotypes with above-average Cu nutritional yield. The second year had unstable, lower than average Cu nutritional yield (score of −6.45) and the first year had the most stable, higher than average Cu nutritional yield (score of 1.74; Figure 2c).

With regard to the genotypes with above-average Mg nutritional yield, the old cultivar Hansa brun showed the most stable Mg nutritional yield (score of 0.008), followed by the landrace Jacoby (score of 0.02) and the old cultivars Walde (score of 0.09) and Starke (score of 0.26). Among the genotypes with lower than average Mg nutritional yield, the most stable was the primitive genotype Svart emmer (score of −0.11; Figure 2d). The most stable genotypes were the ones with higher than average, and even the highest, Mg nutritional yield. The second year of study showed unstable, lower than average Mg nutritional yield (score of −4.76), while the third year had the highest, most stable Mg nutritional yield (score of 0.63; Figure 2d).

A summary of the rankings of the various genotypes from the AMMI stability analysis (i.e., high values of yield and nutritional yield combined with a PC1 value close to 0; Figure 1 and Figure 2) showed the old cultivar Svale to be most highly ranked for the combination of yield and nutritional yields of the four minerals studied (Table 4). The landrace Jacoby was ranked third for yield, second for Fe and Zn nutritional yield, and first for Mg nutritional yield, while the old cultivar Starke ranked first for yield, third for Zn nutritional yield, and second for Cu and Mg nutritional yield. Thus, based on the AMMI stability analysis these genotypes (Svale, Jacoby, and Starke) show the highest yield, nutritional yields, and stability among the 19 winter wheat genotypes studied.

### 3.5. Analysis of Genotypic Value, Stability, and Adaptability for Yield by the BLUP Procedure

The BLUP procedure indicated that the old cultivar Starke had the highest HMGV (4737 kg·ha^−1^), as well as the highest RPGV and HMRPGV (both equal to 1.29; Table 5). Thus, the yield of Starke was simultaneously the highest, the most stable, and the most adaptable to yearly variation of this specific environment. Starke was followed by the old cultivar Svale, with a HMGV of 4439 kg·ha^−1^, a RPGV and a HMRPGV of 1.20; the old cultivar Odin, with a HMGV of 4247 kg·ha^−1^, a RPGV of 1.14, and a HMRPGV of 1.13; the landrace Jacoby, with a HMGV of 4118 kg·ha^−1^, a RPGV and a HMRPGV of 1.13; the cultivar Ure, with a HMGV of 4054 kg·ha^−1^, a RPGV and a HMRPGV of 1.11; and the old cultivar Aura, with a HMGV of 3913 kg·ha^−1^, a RPGV of 1.11 and a HMRPGV of 1.10 (Table 5). All these genotypes, except Aura, also appear at the top of the rankings obtained by the AMMI analysis for yield, confirming that these genotypes are the highest yielding and most stable genotypes for yield.

### 3.6. Analysis of Genotypic Value, Stability, and Adaptability for Fe, Zn, Cu, and Mg Nutritional Yield by BLUP

Ranking of the genotypes as related to nutritional yield of Fe, Zn, Cu, and Mg by the use of the BLUP procedure are shown in Table 6. The HMGV, RPGV, and HMRPGV values for each of the genotypes indicated in general, a high degree of the same genotypes showing high values for nutritional yield of all the four investigated minerals. Thus, the genotypes Svale, Odin, and Starke were among the five genotypes with highest BLUP values for all the four investigated minerals. Furthermore, the genotypes Jacoby and 5113 were among those five showing highest BLUP values for nutritional yield of Zn, Cu, and Mg, while Aura and 5113 showed high values for nutritional yield of Cu and Mg and Walde showed high values for nutritional yield of Fe and Zn.

### 3.7. Ranking of the Winter Wheat Genotypes by Selection Indexes

While applying Elston’s Multiplicative Index (EMI) based on the nutritional yields of the four minerals studied, the genotype ranked highest was the old cultivar Svale, followed by the old cultivar Starke, the landrace Jacoby, and the old cultivars Odin, Walde, and Ertus (Table 7).

The genotype with the highest Baker’s Desired Gains Index (BDGI) including the nutritional yields of the four minerals studied was the old cultivar Walde, followed by the old cultivars Hansa brun, Starke and Svale, the spelt Oberkulmer, and the landrace Jacoby (Table 7).

### 3.8. Contribution to Recommended Daily Intake

If the genotypes in the present study are consumed as whole grain, bread, or flour products, at an average Swedish consumption rate, their contribution to daily requirements is around 15% for whole grain but almost 90% for flour (Table S1). Thus, given the present consumption pattern in Sweden, a high nutrient density in flour is of the outmost importance.

## 4. Discussion

Rankings of the genotypes in the present study resulted in determination of genotypes with high nutritional yields for the four minerals under study that were at stable for that nutritional yield (“balanced” genotypes) across the three years of study (Table 4 and Table 6). The importance of breeding for stability has been stressed earlier [40,53,54] as it is economically essential both for farmers and end-users to have reliable production every year. From the AMMI stability analysis it can be inferred that, in spite of a significant Genotype × Environment interaction effect for nutritional yield, there were genotypes that could confidently be selected combining both stability and high nutritional yield. The BLUP procedure allowed for the identification of genotypes with high genotypic value and high stability/adaptability. Some genotypes appeared at the top of the rankings independent of the applied methods, increasing the confidence that these genotypes should be selected for breeding highly nutritional populations. In fact, stability across environments is a requirement for breeding for high nutrient content to be possible [1]. Thus, “balanced” genotypes with local adaptation, stability, good yields, and high mineral contents can be an option for production in low-input agricultural systems, prevalent in both developed and developing countries.

The present study was able to identify winter wheat genotypes locally adapted to organic agriculture and with notable nutritional concentrations and high nutritional yields for the minerals Fe, Zn, Cu, and Mg (Table 1 and Table 8). The newly introduced metrics of nutritional yield [2] opens up options to compare the usefulness of different production systems for food production to feed the growing population of the world. Therefore, we made a comparison of the nutritional yields obtained from the highest nutritional yielding genotypes in this study with nutritional yields calculated from reports [30,55] on yield and concentration of Fe, Zn, Cu, and Mg in conventional or intensive wheat trials (Table 8). The modern conventional Swedish bread wheat varieties had a yield 1.7 times higher [56] than the genotypes evaluated in the present study. Furthermore, the nutritional yields of the conventional Swedish wheat varieties [56] were 1.46, 1.37, 1.44, and 1.66 times higher for Fe, Zn, Cu, and Mg, respectively, as compared to the wheat from the present study (Table 8). Intensively grown French elite wheat genotypes [30], with a substantially higher yield than the wheat in the present study, resulted in higher nutritional yields despite a low concentration of minerals in the grain. Differences in relationships between yield and mineral content in cereal grains might be related to variations in nitrogen uptake in the cereals. However, nitrogen uptake was not recorded in the present study and is therefore an issue to be evaluated in future studies. If a breeding program could develop novel high yielding genotypes with high concentration of minerals in the grains, this would of course lead to high nutritional yields of such genotypes. However, recent studies have indicated a decrease in nutritional compounds e.g., Fe and Zn in modern varieties, indicating that genetic improvements have resulted in the dilution of nutrition [51]. One breeding program targeting high grain mineral concentration is headed by the Consultative Group for International Agricultural Research (CGIAR). This organization has a specific program (HarvestPlus) with a mission to develop and promote biofortified food crops from staple crops rich in vitamins and minerals (www.harvestplus.org). However, the CGIAR research programs are mainly targeting the poor in developing countries, although knowledge coming from such research programs might also influence the breeding of high-yielding varieties for developed countries. One important aspect to solve with improved biofortification of crops is the increase in phytate, which reduces the bioavailability of Fe and Zn and often goes hand in hand with an increase in these trace elements [51]. A previous study on mineral content in organically grown wheat in Sweden did not show obvious correlations between Fe, Zn, and P (phytate) [3]. The bioavailability of the Fe and Zn was not evaluated in the present investigation and is a matter for future investigations.

The nutrient density was higher in the wheat in the present study as compared to both the conventional Swedish bread wheat varieties and the French elite wheat genotypes. This is clearly seen in the fact that around 270 g of wheat (i.e., of Svale and Walde) are needed in order to achieve 100% of the daily recommended intake (DRI; Table 8). For conventional and elite wheat, more than 300 g, or even more than 350 g, in some cases (Table 8), is needed for the same achievement. Average wheat consumption varies in different countries, but to secure adequate intake of minerals from wheat consumption a nutrient-dense food is desired. Nutrient-dense food [57] has obvious implications for the caloric intake of consumers, which in many occasions is intended to be reduced, particularly in places where obesity and overweight are health issues [25,58]. Thus, taking both nutritional yields and nutrient density into consideration, some of the genotypes evaluated here are clearly alternatives to conventionally produced wheat.

In Nordic countries, cereals are known to contribute a significant part, approx. 90% of Fe, of the daily mineral requirement in the diet [25]. Thus, with the present consumption of whole grain products, only around 15% of the daily requirements of the four evaluated minerals are consumed by the average consumer in Sweden, while the consumption of flour-based products contributes almost 90% of the daily requirement (Table S1). However, these shares of the daily requirements of the four minerals evaluated are reached at consumption of the genotypes in the present study. Less nutrient-dense wheat genotypes than the highest ones evaluated in the present study would contribute even less to fulfilling daily requirements than the genotypes evaluated here. If less nutrient-dense genotypes are to be used (e.g., conventional wheat varieties), recommendations for intake of cereal products need to be increased to reach adequate intake levels of the four evaluated minerals, especially Fe and Zn. As whole grain products are also known as an important source of other bioactive compounds besides the evaluated minerals, it might be desirable to increase the average consumption of whole-grain-based cereal products [3,21,59].

It was noted in the present study that the genotype with the highest yield (Starke) was not the genotype with the highest concentration (expressed in mg·kg^−1^) of the four minerals studied. Conversely, the genotypes with the highest mineral concentrations, such as primitives *T. monococcum* and Svart emmer, did not show high yields. This inverse relationship between yield and mineral concentration has been described in the past [13,14], and in fact low-yielding primitive genotypes produced in organic conditions have been shown to have a higher concentration of many nutritionally important minerals, while high-yielding cultivars have been shown to have lower mineral concentrations [3,14]. What this study has shown is that evaluating genotypes by nutritional yield allows for the selection of more “balanced” genotypes that do not have the maximum yields (Table 4, Table 5 and Table 6) but compensate for that with their mineral concentrations, and this is reflected in their nutritional yield. This approach has been recommended in the face of scarce land resources [2], also taking into consideration that more nutritious crops must also be acceptable to farmers in terms of yield [7,8,9,10]. Furthermore, such “balanced” genotypes with high nutritional yield and high nutrient density are of great relevance both for the developing world in order to obtain sufficient mineral concentrations in their food but also in the developed world to increase mineral intake per calorie.

Given the number of nutrients that are of importance to human health and derived from whole grain cereals [60], it is difficult to evaluate genotypes (for breeding and/or selection) in terms of overall nutritional quality. There are also genetic correlations among these nutrients and other economically important traits making it impossible to consider any of these traits separately [46,61]. Thus, selection indexes have been implemented in order to evaluate genotypes for several traits [46,61]. In this study, we calculated selection indexes to summarize the nutritional yields for which the genotypes in this study were evaluated. Even though the two indexes we employed are conceptually very different [46,49,50], the same genotypes were located in top positions of their respective rankings, i.e., Svale, Starke, Jacoby, and Walde. These same also genotypes appear very stable and adaptable, so the selection indexes support and add value to the selection of these genotypes for the continuation of the breeding process.

The necessity of breeding varieties specifically adapted to organic agriculture has been stated previously [4,10,16,19]. Additionally, the necessity that these varieties include among their characteristics a high nutritional value has also been stated [4,20,60,62,63]. Breeding organic wheat for nutritional value will add to the benefits perceived by consumers regarding the consumption of organic foods [56]. The yields obtained by some old varieties in the present study suggest that these perform well under the conditions of organic agriculture. It may be said that when dealing with breeding for organic agriculture, it is important to select varieties with high nutritional value, high performance against weeds, and high yield, but this kind of comprehensive breeding for organic agriculture is still scarce [4,10]. The present study is part of a bigger project aimed at generating cereal populations adapted to organic production conditions prevalent in Sweden, and with desirable health-promoting, nutritional, and baking characteristics.

## 5. Conclusions

Among the genotypes in the present study, we were able to rank Svale, Starke, Jacoby, and Walde as balanced genotypes of relevance for future breeding for organic cultivation in terms of nutritional yield and nutrient density. These genotypes showed only somewhat lower nutritional yield as compared to conventionally grown genotypes in Sweden, although substantially lower than intensively grown French elite wheat genotypes. However, these genotypes clearly showed higher nutrient density as compared to the two mentioned groups of genotypes. Furthermore, the four most highly ranked genotypes in the present study showed good stability and adaptability to the growing environments in the present study, indicating their local adaptability to organic cultivation in a Swedish environment.

## Figures and Tables

**Table 1:** Summary of weather data recorded in the locality of Ekhaga during the three years of production of 19 winter wheat genotypes. Weather data recorded between May and July of each year are shown in order to highlight the growth period of winter wheat.

Season or Period	Year	Accumulated Precipitation (mm)	Mean Temperature (°C)	Highest Temperature (°C)	Lowest Temperature (°C)	Degree Days (Base 10 °C)
Autumn–Winter	2011–2012	229.5	2.3	5.6	−1.3	52
Spring–Summer	2012	406.0	12.0	17.1	6.8	675
May–July	2012	208.0	13.8	19.0	8.3	412
Autumn–Winter	2012–2013	210.5	−0.3	2.8	−3.8	17
Spring–Summer	2013	212.0	12.7	18.4	6.7	866
May–July	2013	74.5	15.8	21.4	9.7	554
Autumn–Winter	2013–2014	291.0	2.9	5.7	−0.1	29
Spring–Summer	2014	281.0	13.0	18.8	6.9	840
May–July	2014	140.0	14.8	20.3	8.5	519

**Table 2:** Average values (*N* = 6) for yield and nutritional yield of Fe, Zn, Cu and Mg observed in 19 winter wheat genotypes representing six genotype groups across three years of production (2012, 2013, and 2014) under organic conditions in the locality of Ekhaga. Yield values for primitive wheats and spelt wheats were adjusted in order to consider a 25% reduction due to dehulling [32]. Values in parentheses are standard deviations.

ID No.	Genotype Designation	Genotype Group	Yield (kg·ha^−1^)	Nutritional Yield (Adults ha^−1^·Year^−1^)			
				Fe	Zn	Cu	Mg
1	5113	Selection	3807 (942)	39 (12)	47 (9)	65 (12)	47 (15)
4	Borstvete Gotland	Landrace	3619 (1124)	36 (12)	46 (8)	71 (20)	45 (13)
9	Jacoby	Landrace	4595 (1352)	40 (13)	51 (9)	70 (34)	50 (16)
17	Ure	Cultivar	4470 (1196)	37 (14)	42 (13)	57 (16)	48 (20)
10	Oberkulmer	Spelt	3543 (933)	44 (17)	49 (18)	60 (11)	40 (11)
12	Speltvete Gotland	Spelt	2777 (850)	37 (13)	46 (13)	58 (17)	34 (10)
15	Svart emmer	Primitive	2525 (1227)	34 (20)	45 (23)	50 (24)	33 (17)
16	*T. monococcum*	Primitive	2027 (1115)	28 (18)	43 (25)	51 (30)	25 (15)
2	Aros	Old cultivar	4369 (1319)	34 (11)	42 (11)	64 (13)	45 (17)
3	Aura	Old cultivar	4520 (1673)	37 (20)	45 (16)	64 (19)	50 (22)
5	Erbe	Old cultivar	3938 (1614)	34 (15)	42 (14)	65 (20)	47 (25)
6	Ertus	Old cultivar	4063 (1860)	41 (19)	46 (20)	66 (30)	44 (22)
7	Hansa brun	Old cultivar	4269 (1044)	46 (19)	46 (13)	58 (7)	46 (14)
8	Holger	Old cultivar	3754 (1540)	34 (14)	44 (18)	54 (16)	44 (20)
11	Odin	Old cultivar	4572 (982)	41 (10)	47 (8)	79 (14)	47 (14)
13	Starke	Old cultivar	5307 (1335)	42 (17)	52 (9)	74 (12)	56 (19)
14	Svale	Old cultivar	4912 (1204)	54 (14)	48 (10)	75 (12)	53 (18)
18	Vakka	Old cultivar	3875 (1602)	33 (12)	40 (13)	51 (29)	46 (22)
19	Walde	Old cultivar	4315 (1318)	44 (14)	52 (16)	64 (13)	45 (17)

**Table 3:** Pearson correlation coefficients among yield and nutritional yield for Fe, Zn, Cu, and Mg.

Source	Yield	Fe Nutritional Yield	Zn Nutritional Yield	Cu Nutritional Yield
Fe nutritional yield	0.80 ***			
Zn nutritional yield	0.72 ***	0.78 ***		
Cu nutritional yield	0.76 ***	0.71 ***	0.73 ***	
Mg nutritional yield	0.95 ***	0.84 ***	0.76 ***	0.76 ***

*** = Significant at *p* < 0.005.

**Table 4:** Rankings of the winter wheat genotypes with the highest yield or nutritional yield and stability for Fe, Zn, Cu, and Mg according to AMMI stability analysis (i.e., combined high values of yield and nutritional yield with PC1 values close to 0; Figure 1 and Figure 2). The genotype groups to which the genotypes belong are shown in parenthesis. Genotypes sharing a ranking could not be differentiated from each other according to nutritional yield and stability scores.

Rank	Yield (kg·ha^−1^)	Nutritional Yield (Adults ha^−1^·Year^−1^)			
		Fe	Zn	Cu	Mg
1	Starke (OC)	Walde (OC)	Svale (OC)	Svale (OC)	Jacoby (LR), Svale (OC)
2	Svale (OC), Ure (C)	Jacoby (LR)	Jacoby (LR)	Starke (OC)	Starke (OC)
3	Jacoby (LR), Walde (OC)	Svale (OC)	Starke (OC)	Aros (OC), Walde (OC)	Odin (OC)
4	Odin (OC)	Aros(OC)	Walde (OC)	Odin (OC)	Walde (OC)
5	Aros (OC), Hansa brun (OC)	Odin (OC)	5113 (S)	5113 (S)	Ure (C)
6	Erbe (OC)	5113 (S), Hansa brun (OC), Ure (C)	Speltvete Gotland (SP)	Erbe (OC)	5113 (S), Aura (OC)

C = cultivar, LR = Landrace, OC = Old cultivar, S = Selection, SP = Spelt.

**Table 5:** Rankings of the winter wheat genotypes with the highest, most adaptable, and most stable yield according to genotypic values (BLUP). Values shown are the harmonic mean of genotypic values (HMGV), which illustrates stability; the relative performance of genotypic values (RPGV), which represents adaptability to unfavorable conditions; and the harmonic mean of relative performance of genotypic values (HMRPGV), which simultaneously denotes high yield, stability, and adaptability.

Rank	Genotype	Genotype Group	HMGV (kg·ha^−1^)	RPGV	HMRPGV
1	Starke	Old cultivar	4737	1.29	1.29
2	Svale	Old cultivar	4439	1.20	1.20
3	Odin	Old cultivar	4247	1.14	1.13
4	Jacoby	Landrace	4118	1.13	1.13
5	Ure	Cultivar	4054	1.11	1.11
6	Aura	Old cultivar	3913	1.11	1.10

**Table 6:** Rankings of the winter wheat genotypes with the highest, most adaptable, and most stable nutritional yield of Fe, Zn, Cu, and Mg according to genotypic values (BLUP). Values shown are the harmonic mean of genotypic values (HMGV), which illustrates stability; the relative performance of genotypic values (RPGV), which represents adaptability to unfavorable conditions; and the harmonic mean of relative performance of genotypic values (HMRPGV), which simultaneously denotes high nutritional yield, stability, and adaptability.

Rank	Genotype	Genotype Group	HMGV (Adults ha^−1^·Year^−1^)	RPGV	HMRPGV
**Fe Nutritional Yield**					
1	Svale	Old cultivar	46	1.29	1.28
2	Hansa brun	Old cultivar	39	1.15	1.12
3	Walde	Old cultivar	38	1.08	1.08
4	Oberkulmer	Spelt	39	1.12	1.08
5	Odin	Old cultivar	37	1.05	1.04
6	Starke	Old cultivar	36	1.05	1.02
**Zn Nutritional Yield**					
1	Walde	Old cultivar	48	1.09	1.09
2	Starke	Old cultivar	48	1.09	1.08
3	Jacoby	Landrace	47	1.07	1.07
4	Svale	Old cultivar	45	1.03	1.03
5	Odin	Old cultivar	46	1.03	1.02
6	5113	Selection	45	1.02	1.01
**Cu Nutritional Yield**					
1	Odin	Old cultivar	71	1.16	1.15
2	Svale	Old cultivar	69	1.13	1.13
3	Starke	Old cultivar	68	1.11	1.11
4	Jacoby	Landrace	62	1.07	1.05
5	5113	Selection	62	1.02	1.01
6	Aura	Old cultivar	61	1.02	1.01
**Mg Nutritional Yield**					
1	Starke	Old cultivar	46	1.20	1.20
2	Svale	Old cultivar	44	1.15	1.15
3	Jacoby	Landrace	42	1.09	1.09
4	Aura	Old cultivar	39	1.08	1.07
5	Odin	Old cultivar	42	1.07	1.06
6	5113	Selection	41	1.05	1.05

**Table 7:** Rankings of the winter wheat genotypes with the highest Elston multiplicative selection index (EMI) and Baker’s desired gains index (BDGI). The genotype group to which each genotype belongs is also shown.

Rank	EMI		BDGI	
	Genotype	Genotype Group	Genotype	Genotype Group
1	Svale	Old cultivar	Walde	Old cultivar
2	Starke	Old cultivar	Hansa brun	Old cultivar
3	Jacoby	Landrace	Starke	Old cultivar
4	Odin	Old cultivar	Svale	Old cultivar
5	Walde	Old cultivar	Oberkulmer	Spelt
6	Ertus	Old cultivar	Jacoby	Landrace

**Table 8:** Comparison of concentration (mg·kg^−1^), nutritional yield (adults ha^−1^·year^−1^), and the amount (g) that must be consumed in order to achieve 100% of the daily recommended intake [25] of Fe, Zn, Cu, and Mg, among selected genotypes evaluated in this study and wheat genotypes studied under conventional conditions in Sweden [54] or intensive conditions in France [30].

Genotypes (or Location)	Yield (kg·ha^−1^)	Concentration (mg·kg^−1^)				Nutritional Yield (Adults ha^−1^·Year^−1^)				Amount to be Consumed to Achieve 100% DRI (g)				Reference
		Fe	Zn	Cu	Mg	Fe	Zn	Cu	Mg	Fe	Zn	Cu	Mg	
Akteur	6920	38.0	26.4	3.8	1220	60	63	80	73	316	303	236	258	Lundegårdh et al. (2009) [55]
Olivin	7180	34.7	23.9	4.0	1260	57	59	87	79	346	335	226	250	
(Fransåker)	8190	39.8	29.3	4.6	1110	74	82	115	79	302	273	194	284	
Elite modern (CF)	7510	33.1	17.3	N/A	1109	57	44	N/A	72	363	462	N/A	284	Oury et al. (2006) [30]
Elite modern (LM)	10780	39.1	22.9	N/A	1006	96	85	N/A	94	307	349	N/A	313	
Elite modern (RE)	11560	31.9	21.2	N/A	894	84	84	N/A	90	376	377	N/A	352	
Svale	4912	49.0	29.0	5.1	1202	54	48	75	53	245	276	176	262	This study
Starke	5307	34.6	29.2	4.7	1185	42	52	74	56	347	274	191	266	
Jacoby	4595	38.2	33.9	4.7	1225	40	51	70	50	314	236	191	257	
Walde	4315	43.9	36.4	5.1	1181	44	52	64	45	273	220	176	267	
Oberkulmer	3543	52.9	39.9	5.6	1302	44	49	60	40	227	201	161	242	
Speltvete Gotland	2777	58.0	50.0	7.1	1399	37	46	58	34	207	160	127	225	

CF = Clermon Ferrand, LM = Le Moulon, RE = Rennes, DRI = Daily Recommended Intake.

## Supplementary Materials

The following are available online at www.mdpi.com/2304-8158/5/4/89/s1, Table S1: Average values of percentage of contribution to the daily recommended intake of Fe, Zn, Cu, and Mg [23] by 19 winter wheat genotypes representing six genotype groups, considering the Swedish average consumption of whole grain (42.5 g·day^−1^) [48], bread (86 g·day^−1^) [55], and wheat flour (200 g·day^−1^).

## References

[1] Welch R.M. (2002). The impact of mineral nutrients in food crops on global human health. Plant Soil.

[2] DeFries R., Fanzo J., Remans R., Palm C., Wood S., Anderman T.L. (2015). Metrics for land-scarce agriculture. Science.

[3] Hussain A., Larsson H., Kuktaite R., Johansson E. (2010). Mineral composition of organically grown wheat Genotypes: Contribution to daily minerals intake. Int. J. Environ. Res. Public Health.

[4] Lammerts van Bueren E.T., Jones S.S., Tamm L., Murphy K.M., Myers J.R., Leifert C., Messmer M.M. (2011). The need to breed crop varieties suitable for organic farming, using wheat, tomato and broccoli as examples: A review. NJAS Wageningen J. Life Sci..

[5] Ortiz-Monasterio J.I., Palacios-Rojas N., Meng E., Pixley K., Trethowan R., Peña R.J. (2007). Enhancing the mineral and vitamin content of wheat and maize through plant breeding. J. Cereal Sci..

[6] Graham R.D., Welch R.M., Bouis H.E. (2001). Addressing micronutrient malnutrition through enhancing the nutritional quality of staple foods: Principles, perspectives and knowledge gaps. Adv. Agron..

[7] Welch R.M., Graham R.D. (2004). Breeding for micronutrients in staple food crops from a human nutrition perspective. J. Exp. Bot..

[8] Drewnowski A. (2005). Concept of a nutritious food: Towards a nutrient density score. Am. J. Clin. Nutr..

[9] Welch R.M., Graham R.D. (2002). Breeding crops for enhanced micronutrient content. Plant Soil.

[10] Pfeiffer W.H., McClafferty B. (2007). HarvestPlus: Breeding crops for better nutrition. Crop Sci..

[11] White P.J., Broadley M.R. (2005). Biofortifying crops with essential mineral elements. Trends Plant Sci..

[12] White P.J., Broadley M.R. (2009). Biofortification of crops with seven mineral elements often lacking in human diets—Fe, Zn, Cu, calcium, Mg, selenium and iodine. New Phytol..

[13] Murphy K.M., Reeves P.G., Jones S.S. (2008). Relationship between yield and mineral nutrient concentrations in historical and modern spring wheat cultivars. Euphytica.

[14] Fan M.-S., Zhao F.-J., Fairweather-Tait S.J., Poulton P.R., Dunham S.J., McGrath S.P. (2008). Evidence of decreasing mineral density in wheat grain over the last 160 years. J. Trace Elem. Med. Biol..

[15] Schmidhuber J., Tubiello F.N. (2007). Global food security under climate change. PNAS.

[16] Wolfe M.S., Baresel J.P., Desclaux D., Goldringer I., Hoad S., Kovacs G., Löschenberger F., Miedaner T., Østergård H., van Bueren E.T.L. (2008). Developments in breeding cereals for organic agriculture. Euphytica.

[17] Godfray H.C.J., Beddington J.R., Crute I.R., Haddad L., Lawrence D., Muir J.F., Pretty J., Robinson S., Thomas S.M., Toulmin C. (2010). Food security: The challenge of feeding 9 billion people. Science.

[18] Hussain A. (2012). Quality of Organically Produced Wheat from Diverse Origin. Ph.D. Thesis.

[19] Phillips S.L., Wolfe M.S. (2005). Evolutionary plant breeding for low input systems. J. Agric. Sci..

[20] Hussain A., Larsson H., Kuktaite R., Johansson E. (2012). Healthy food from organic wheat: Choice of genotypes for production and breeding. J. Sci. Food Agric..

[21] Hussain A., Larsson H., Olsson M.E., Kuktaite R., Grausgruber H., Johansson E. (2012). Is organically produced wheat a source of tocopherols and tocotrienols for health food?. Food Chem..

[22] Johansson E., Hussain A., Kuktaite R., Andersson S.C., Olsson M.E. (2014). Contribution of organically grown crops to human health. Int. J. Environ. Res. Public Health..

[23] (2004). International Zinc Nutrition Consultative Group (IZiNCG) Assessment of the risk of zink deficiency in populations and options for its control. Food Nutr. Bull..

[24] Spiegel H., Sager M., Oberforster M., Mechtler K., Stüger H.P., Baumgarten A. (2009). Nutritionally relevant elements in staple foods: Influence of arable site versus choice of variety. Environ. Geochem. Health.

[25] Nordic Council of Ministers (2012). Nordic Nutrition Recommendations 2012: Integrating Nutrition and Physical Activity.

[26] Monasterio I., Graham R.D. (2000). Breeding for trace minerals in wheat. Food Nutr. Bull..

[27] Xu Y., An D., Li H., Xu H. (2011). Review: Breeding wheat for enhanced micronutrients. Can. J. Plant Sci..

[28] Bouis H.E., Welch R.M. (2010). Biofortification—A sustainable agricultural strategy for reducing micronutrient malnutrition in the Global South. Crop Sci..

[29] Martínez-Ballesta M.C., Dominguez-Perles R., Moreno D.A., Muries B., Alcaraz-López C., Bastías E., García-Viguera C., Carvajal M. (2010). Minerals in plant food: Effect of agricultural practices and role in human health. A review. Agron. Sustain. Dev..

[30] Oury F.-X., Leenhardt F., Rémésy C., Chanliaud E., Duperrier B., Balfourier F., Charmet G. (2006). Genetic variability and stability of grain Mg, Zn and Fe concentrations in bread wheat. Eur. J. Agron..

[31] Hussain A., Larsson H., Kuktaite R., Johansson E. (2012). Concentration of some heavy metals in organically grown primitive, old and modern wheat genotypes: Implications for human health. J. Environ. Sci. Health B.

[32] Stallknecht G.F., Gilbertson K.M., Ranney J.E., Janick J. (1996). Alternative Wheat Cereals as Food Grains: Einkorn, Emmer, Spelt, Kamut, and Triticale.

[33] Malik A.H., Kuktaite R., Johansson E. (2013). Combined effect of genetic and environmental factors on the accumulation of proteins in the wheat grain and their relationships to bread-making quality. J. Cereal Sci..

[34] R Development Core Team (2012). R: A Language and Environment for Statistical Computing.

[35] Vagiri M., Ekholm A., Öberg E., Johansson E., Andersson S.C., Rumpunen K. (2013). Phenols and ascorbic acid in black currants (*Ribes nigrum* L.): Variation due to genotype, location and year. J. Agric. Food Chem..

[36] Andersson S.C., Olsson M.E., Gustavsson K.-E., Johansson E., Rumpunen K. (2012). Tocopherols in rose hips (*Rosa* spp.) during ripening. J. Sci. Food Agric..

[37] Andersson S.C., Rumpunen K., Johansson E., Olsson M.E. (2008). Tocopherols and tocotrienols in Sea Buchthorn (*Hoppophae rhamnoides*) berries during ripening. J. Agric. Food Chem..

[38] Johansson E., Prieto-Linde M.-L., Svensson G., Jönsson J. (2003). Influences of cultivar, cultivation year and fertilizer rate on amount of protein groups and amount and size distribution of mono- and polymeric proteins. J. Agric. Sci..

[39] Johansson E., Prieto-Linde M.-L., Gissén C. (2008). Influences of weather, cultivar and fertilizer rate on grain protein accumulation in field-grown wheat, and relations to grain water content and falling number. J. Sci. Food Agric..

[40] Annicchiarico P. (2002). Genotype × Environment Interactions—Challenges and Opportunities for Plant Breeding and Cultivar Recommendations.

[41] Annicchiarico P. (1997). Additive main effects and multiplicative interaction (AMMI) analysis of genotype-location interaction in variety trials repeated over years. Theor. Appl. Genet..

[42] Gauch H.G., Piepho H.-P., Annicchiarico P. (2008). Statistical analysis of yield trials by AMMI and GGE: Further considerations. Crop Sci..

[43] Gauch H.G., Zobel R.W. (1988). Predictive and postdictive success of statistical analyses of yield trials. Theor. Appl. Genet..

[44] Gauch H.G. (1992). Statistical Analysis of Regional Yield Trials: AMMI Analysis of Factorial Design.

[45] Agricolae: Statistical Procedures for Agricultural Research. http://cran.r-project.org/web/packages/agricolae/index.html.

[46] Bernardo R. (2002). Breeding for Quantitative Traits in Plants.

[47] De Resende M.D.V. (2004). Métodos Estatísticos Ótimos na Análise de Experimentos de Campo.

[48] Bates D., Mächler M., Bolker B., Walker S. (2015). Fitting linear mixed-effects models using lme4. J. Stat. Softw..

[49] Elston R.C. (1963). A Weight-free index for the purpose of ranking or selection with respect to several traits at a time. Biometrics.

[50] Pesek J., Baker R.J. (1970). An application of index selection to the improvement of self-pollinated species. Can. J. Plant Sci..

[51] Zhao F.J., Su Y.H., Dunham S.J., Rakszegi M., Bedo Z., McGrath S.P., Shewry P.R. (2009). Variation in mineral micronutrient concentrations in grain of wheat lines of diverse origin. J. Cereal Sci..

[52] Graham R.D., Welch R.M., Saunders D.A., Ortiz-Monasterio I., Bouis H.E., Bonierbale M., de Haan S., Burgos G., Thiele G., Liria R., Sparks D.L. (2007). Nutritious Subsistence Food Systems.

[53] Kang M.S. (1993). Simultaneous selection for yield and stability in crop performance trials: Consequences for growers. Agron. J..

[54] Johansson E., Svensson G., Tsegaye S. (2000). Genotype and environment effects on bread-making quality of Swedish grown wheat cultivars containing HMW glutenin subunits 2 + 12 and 5 + 10. Acta Agric. Scand..

[55] Lundegårdh B., Jastrebova J., Zhokhov S., Mårtensson A., Öborn I. (2009). Effects of Growing Location and Variety on Free Tryptophan and Mineral Nutrient Content in Wheat.

[56] Seufert V., Ramankutty N., Foley J.A. (2012). Comparing the yields of organic and conventional agriculture. Nature.

[57] Nicklas T.A., Drewnowski A., O’Neil C.E. (2014). The nutrient density approach to healthy eating: Challenges and opportunities. Public Health Nutr..

[58] Frølich W., Åman P., Tetens I. (2013). Whole grain foods and health—A Scandinavian perspective. Food Nutr. Res..

[59] Hussain A., Larsson H., Kuktaite R., Olsson M.E., Johansson E. (2015). Carotenoid content in organically produced wheat: Relevance for human nutritional health on consumption. Int. J. Environ. Res. Public Health.

[60] Fardet A. (2010). New hypotheses for the health-protective mechanisms of whole-grain cereals: What is beyond fibre?. Nutr. Res. Rev..

[61] Geraldi I. (2005). Selection Indices for Population Improvement Programmes.

[62] Fardet A. (2014). How can both the health potential and sustainability of cereal products be improved? A French perspective. J. Cereal Sci..

[63] Van Bueren E.T.L., Struik P.C., Tiemens-Hulscher M., Jacobsen E. (2003). Concepts of intrinsic value and integrity of plants in organic plant breeding and propagation. Crop Sci..

