# Supplementary material for: Mineral Nutritional Yield and Nutrient Density of Locally Adapted Wheat Genotypes under Organic Production
Source: Foods. 2016 Dec 20;5(4):89. doi: 10.3390/foods5040089 (PMC5302432; doi:10.3390/foods5040089)
Supplement: Supplementary file 1 [file foods-05-00089-s001.pdf]

Supplementary Materials: Mineral Nutritional Yield and Nutrient Density of Locally Adapted Wheat Genotypes under Organic Production

Sergio Daniel Moreira-Ascarrunz, Hans Larsson, Maria Luisa Prieto-Linde and Eva Johansson

Table S1. Average values of percentage of contribution to the daily recommended intake of Fe, Zn, Cu, and Mg [1] by 19 winter wheat genotypes, representing six genotype groups. In these calculations we have applied Swedish average consumption levels similar to those applied in previous publications for whole grain (42.5 g·day^{-1}) [2], bread (86 g·day^{-1}) [3], and wheat flour (200 g·day^{-1}) products [4].

ID No.	Genotype Designation	Whole Grain Cereals				Bread				Flour			
		Fe (%)	Zn (%)	Cu (%)	Mg (%)	Fe (%)	Zn (%)	Cu (%)	Mg (%)	Fe (%)	Zn (%)	Cu (%)	Mg (%)
1	5113	15.7	19.7	27.1	18.8	31.8	39.9	54.8	38.0	74.0	92.9	127.5	88.5
2	Aros	12.2	15.3	23.5	15.4	24.7	30.9	47.6	31.1	57.4	72.0	110.8	72.3
3	Aura	11.4	15.8	22.9	16.6	23.1	31.9	46.4	33.5	53.7	74.1	107.9	78.0
4	Borstvete Gotland	15.5	20.8	31.0	19.4	31.4	42.1	62.7	39.3	73.0	97.8	145.8	91.5
5	Erbe	13.3	17.3	26.8	17.4	26.8	34.9	54.2	35.1	62.4	81.2	125.9	81.7
6	Ertus	16.0	18.1	26.2	16.4	32.3	36.7	53.0	33.3	75.1	85.3	123.3	77.4
7	Hansa brun	16.4	16.8	21.7	16.4	33.2	34.1	43.9	33.2	77.3	79.3	102.0	77.2
8	Holger	14.0	18.6	24.2	17.8	28.4	37.7	48.9	36.0	66.1	87.7	113.7	83.7
9	Jacoby	13.5	18.0	22.2	16.5	27.4	36.4	45.0	33.4	63.7	84.7	104.5	77.8
10	Oberkulmer	18.7	21.2	26.7	17.6	37.9	42.9	54.0	35.5	88.1	99.7	125.6	82.7
11	Odin	13.8	16.3	27.6	15.8	28.0	33.0	55.9	32.1	65.1	76.7	130.1	74.6
12	Speltvete Gotland	20.6	26.6	33.4	18.9	41.6	53.7	67.6	38.2	96.8	125.0	157.3	88.8
13	Starke	12.3	15.5	22.2	16.0	24.8	31.4	45.0	32.4	57.7	73.0	104.7	75.3
14	Svale	17.3	15.4	24.3	16.2	35.1	31.2	49.1	32.8	81.6	72.5	114.2	76.3
15	Svart emmer	20.1	27.3	31.3	20.1	40.6	55.3	63.4	40.6	94.5	128.7	147.5	94.4
16	*Triticum monococcum*	19.9	32.6	39.5	18.8	40.3	66.0	80.0	38.1	93.7	153.5	186.0	88.5
17	Ure	12.6	14.7	20.3	16.0	25.4	29.7	41.0	32.4	59.1	69.1	95.4	75.3
18	Vakka	13.8	16.9	22.6	17.6	27.9	34.2	45.7	35.7	64.8	79.6	106.3	82.9
19	Walde	15.6	19.3	24.0	15.9	31.5	39.1	48.5	32.2	73.2	91.0	112.8	75.0

References

1. International Zinc Nutrition Consultative Group (IZiNCG) Assessment of the risk of zink deficiency in populations and options for its control. *Food Nutr. Bull.* **2004**, *25*, S91–S204.
2. Frølich, W.; Åman, P.; Tetens, I. Whole grain foods and health—A Scandinavian perspective. *Food Nutr. Res.* **2013**, *57*, doi:10.3402/fnr.v57i0.18503.
3. Sandvik, P.; Kihlberg, I.; Lindroos, A.K.; Marklinder, I.; Nydahl, M. Bread consumption patterns in a Swedish national dietary survey focusing particularly on whole-grain and rye bread. *Food Nutr. Res.* **2014**, *58*, doi:10.3402/fnr.v58.24024.
4. Hussain, A.; Larsson, H.; Kuktaite, R.; Johansson, E. Mineral composition of organically grown wheat Genotypes: Contribution to daily minerals intake. *Int. J. Environ. Res. Public Health* **2010**, *7*, 3442–3456.
